# Mitigating effects of Jambul against lead induced toxicity in epididymis and vas deferens of mice

**Published:** 2015-11

**Authors:** Tahir Abbas, Khawaja Raees Ahmad, Asmat Ullah, Samreen Iqbal, Kausar Raees

**Affiliations:** 1 *Department of Biology, Government Degree College, Kotmomin Sargodha, Pakistan.*; 2 *University of Sargodha, Sargodha, Pakistan. *; 3 *University of the Punjab Lahore, Pakistan.*; 4 *Department of Biology, Government College for Women Farooq Colony Sargodha, Pakistan.*

**Keywords:** *Epididymis*, *Vas deferens*, *Apoptosis*, *Stereocilia*, *Jambul*

## Abstract

**Background:**

Precious fruits like jambul are neglected and wasted while environmental pollutants like lead intake remain overlooked.

**Objective:**

The aim of this study was to investigate the effects of the Jambul pulp extract on lead detrimental effects in pseudostratified epithelium and the stereocilia of mice epididymis and vas deferens.

**Materials and Methods:**

Thirty young males mice (Mus musculus) were distributed randomly in 3 groups (n= 10) called control, Pb (Lead) and Pb-J (Lead-Jambul). The Pb and Pb-J were provided 50ppm Pb in drinking water ad libitum for 15 days and Pb free water for the next 5 days. The Pb-J group received 0.2ml jambul pulp extract on 12 hourly bases. Control group was not given any treatment. Organs (epididymis and vas deference) were recovered on 21^st^ day after euthanasia. The organs were finally processed for histological and micrometric studies.

**Results:**

Marked histologic and micrometric changes in both organs were noted in Pb group. These include significant (P ≤ 0.05) decrease in cross sectional area of caput and cauda epididymis folding tubing along with evident alterations of their endothelial thickness. Prominent signs of apoptosis (vacuolations) in the corpus pseudostratified endothelium and the destruction of stereocilia of the epididymis and vas deferens in Pb compared to control group were observed. Evident signs of recovery, in both organs, such as proliferation and rearrangements in pseudostratified endothelium and the stereocilia along with convincing recovery in micrometric parameters were observed in Pb-J group.

**Conclusion:**

The results indicate that epididymis and vas deferens are highly sensitive to Pb exposure while Jambul pulp extract has shown rich mitigating potentials against such histopathologies.

## Introduction

Natural environment provides all the basic human needs, but industrial waste and sewage runoff with heavy metals contamination in aquatic ecosystem have increased severe problems (1-2). Heavy metals have cumulative deleterious effects, because of their dense, ductile, malleable and corrosion resistant possession (3). Lead (Pb) has been one of them frequently used it in paints, insecticides, petroleum refining and bullets of gun from there rapidly reached in the blood through inhalation and ingestion (4). Its bioaccumulation is known to potentiate the toxicity leading to histological, immunological, renal, hepatic and testicular anomalies (5-6). Pb oxidative stress induces apoptosis and necrosis of biological membranes and extracellular matrix to promote lipid peroxidation and fatty acid degeneration (7). Lipid peroxidation causes impairment in folliculogenesis and atresia of ovarian follicles (8). It also deposits in the walls of the seminiferous tubules and suppressed the hypothalamic-pituitary-testicular axis to cause testicular histological alterations, reduction in epididymis sperm cells and testosterone (9). 

Black and green tea are the most commonly consumed beverages and are rich in polyphenol compounds similarly black pepper (Piper nigrum) seeds have radical scavenging activity and their antioxidant activity is mainly due to hydrogen donors, singlet oxygen quenchers, metal chelators and reductants of ferryl hemoglobin (10-12). Medicinal plants due to the presence of alkaloids, flavonoids, tannins, and steroids are directed to prevent oxidative damage and have received attention for their potential role in prevention of diseases (13-15).

Syzygium cumini (Jambul, Jambolan, Jamblang, or Jamun) is an evergreen tropical tree is native to the Indian Subcontinent and adjoining regions of Southeast Asia. It belongs to family Myrtaceae and its fruit pulp has excellent antioxidant properties, seeds have free radicals scavenging properties and are source of raffinose, glucose, fructose, citric acid, mallic acid, gallic acid, anthocyanins, delphinidin-3-gentiobioside, cyanidin diglycoside, petunidin and malvidin. Its stem bark is rich in betulinic acid, friedelin, epi-friedelanol, β-sitosterol, eugenin, and fatty acid esters of epi-friedelanol used for cough, dysentery, and chronic diarrhea (16-19).

The combined effects of Pb and Jambul on reproductive organs, especially the epididymis and vas deference, a site of sperm maturation, are not fully known. Therefore, the present investigation was undertaken to study the toxic effects of Pb exposure in mice and the effects of subsequent withdrawal of treatment and recovery on jambul pulp extract post treatment.

## Materials and methods

This experimental study was conducted on the Swiss Webster strain of albino laboratory mice (Mus musculus) under the administrative and ethical control of “Higher studies and Research Board” University of Sargodha, Sargodha Pakistan and University of the Punjab, Lahore Pakistan respectively. These animals were kept in the animal house established in The Department of Zoology, University of Sargodha. On the day 30^th^ after birth, the mice were weaned, housed one male with 2 females in a steel cage (12″ X 18″) to raise the colony of about same age/weight, under standard conditions of 12-hour day/night cycles, at 25±2^o^C and had free access to the rodent diet pellet and tap water. Jambul (Pericarp and bark of jambul are recognized in Pharmacopeia of The Netherlands while the medicinal importance of its ingredients has been recognized by Rainshadowlabs and The UpJohn company) fresh fruit were taken from the market and seed free pulp was grinded to obtain the extract by using an electric blender. Later on the extract was centrifuged to remove fibrous contents and only supernatant was stored at -30^o^C for animal treatments. For getting 50 ppm Pb solution a PbCH3COO (1000ppm) stock solution was prepared by dissolving 2.73g of PbCH3COO/L of water and diluted to get 50ppm Pb required solution. Animals randomly were grouped (n=10) as: Cont; control given food and regular Pb free drinking water, Pb and Pb-J were given 50ppm Pb in drinking water for 15 days, while Pb-J group was additionally given 0.2ml/12h jambul pulp extract for next 5days while both Pb and Pb-J groups were supplied Pb free water for this duration. 

No animal loss and/ or surgical complications occurred during the study period.

On the 21^st^ day of respective treatments all animals were sacrificed by cervical dislocation, epididymis and vas deference were excised. The organs were processed for histology (H & E) and for micrometrical calculations as standard protocol (20-21). Photomicrographs by Sony Model No. DSC-W35 7.2 mega pixel digital camera affixed on a Labomid CXR2 trinocular microscope of 10 randomly selected sections at 100× and 400× from each group were used for micrometric data. Selected photographs were processed in CorelDRAW11 for brightness, sharpness, and contrast. These were finally cropped digitally for final presentation in the result section.

**Statistical analysis**

The micrometrical data were being pooled to obtain mean values in such a way that each animal represented as a unit and was further analyzed on ANOVA and Duncan’s Multiple Range Test, employing Microsoft Excel and Statistical Package for the Social Sciences, version 20.0, SPSS Inc, Chicago, Illinois, USA (SPSS software).

## Results

**Figure 1:**
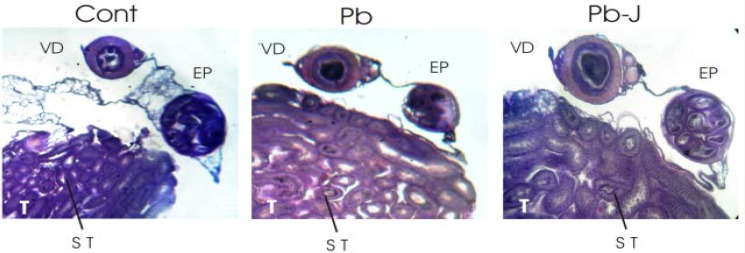
Testis (T), epididymis (EP) and vas deference (VD) in control (Cont) Lead (Pb) and Lead–Jambul (Pb-J) group mice (40×).

The histology of reproductive parts at 100× indicated the proper position of testes, epididymis and vas deference. The epididymis is single narrow, tightly coiled tube in the male reproductive system, which connects the efferent ducts from the rear of each testis to the vas deferens (Figure 1).

**Caput epididymis histology**

**Figure 2:**
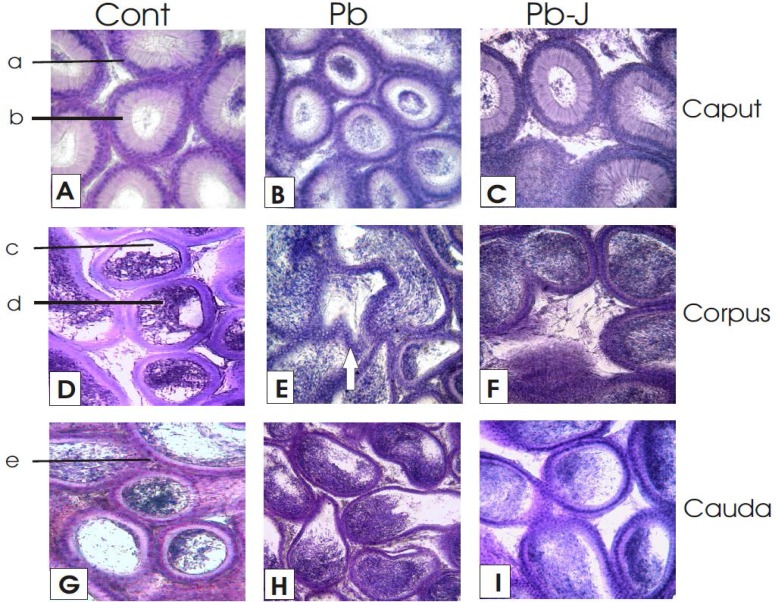
Histological sections of three distinct segments (caput, corpus and cauda) of epididymis in control and experimental group (100×)

The caput epididymis at 400× of control mice showed almost circular sections of compactly arranged folding epididymis tubing containing pseudostratified endothelial cells lined with elongated stereocilia projecting into the spermatozoa filled luminal space (Figure 2-A). The tubular principal cells were more columnar in nature, the muscular layer around the epididymis folding tubing and basal cells with rounded nuclei present almost on the basement membrane. The folding tubing histological sections were more tightly packed, distributed evenly in the epididymis connective tissue (Figure 2-A).

In contrast to the control group, caput region folding epididymis tubing of Pb exposed group showed wider luminal space found choked with dead sperm cells and debris. The tightly packed principal cells, all with basal nuclei, forming endothelium were cuboidal in shape. Some darker bodies were also visible in the lumen of folding tubing, representing the phagocytosis of debris by a presumable macrophages infestation. The stereocilia were less prominent as compared to the control group. The inter-tubular spaces were wider than control (Figure 2-B).

The histological architecture of the caput epididymis in Pb-J group reiterated control group however the inter-tubular spaces were still wider than control (Figure 2-C).

The micrometric observations indicated significant (p≤0.05) reduction of the CSA of caput epididymis folding tubing as compared to other groups while highest epithelial 

thickness/height of caput epididymis folding tubing was noticed in Pb followed by Pb-J and least in C treated group (Table I).

**Corpus epididymis histology**

The histological outlay in control group showed closely packed rounded tubing folding- with cuboidal endothelium and spermatozoa filled luminal spaces (Figure 2-D). In Pb group the tubing folding appeared more elongated and less circular in shape showing various bulging folding and wide spaces in between. Clumping of stereocilia, pyknosis of endothelial cell nuclei with cytoplasmic vacuolations and increased density of sperm and debris were seen than the control group. The disorganized boundary in some tubing marked by arrow head indicated apoptosis in principal endothelial cells along with poorly developed stereocilia and microvilli. The muscular layer around the tubules was far less developed as compared to control (Figure 2-E). In Pb-J group the gross microanatomy of the folding tubing was like that of control group however the luminal space was found almost completely chocked with dead spermatic debris. The inter-tubular fibrosis was mimicking the similar disposition in the Pb group (Figure 2-F). Cross-sectional area of corpus epididymis folding tubing was highest in Pb followed by C and Pb-J group; while highest epithelial height/thickness of corpus epididymis folding tubing from highest to lowest in descending order was observed in C, Pb-J and Pb exposed group (Table I).

**Caudal epididymis histology**

In control group, the folding tubing showed less tall columnar endothelial cells and shorter stereocilia than the caput region. The lumen showed cavities and clumped spermatozoa clinked with a tubular wall at various places. Various clumped masses of the spermatozoa were also visible in the lumen with wide empty spaces in between. The connective tissues around the folding tubing were well developed (Figure 2-G). In Pb group the connective tissues were less dense and folding tubing was irregular (more or less oval shape) with wavy and somewhat diffused endothelial lining with scanty stereocillia. Spermatic mass was seen clumped at the wider ends of the oval sections (Figure 2-H). Cauda epididymis in Pb-J group showed cuboidal principal cells with centrally placed nuclei. The thick muscular layer around tubules and lumen containing free floating spermatozoa and the debris clinked on the wall at one or more places. The stereocilia in cauda regions remained almost similar to that of Pb. The absence of wave like irregular margins (seen in Pb group) and proper circular appearance indicated the sign of recovery jambul pulp induced recovery in this group (Figure 2-I).

Statistical analysis of the data showed significant (P≤0.05) reduction of the cross-sectional area of the cauda epididymis folding tubing in Pb exposed group as compared to control group while the epithelial thickness of the cauda epididymis folding tubing remained almost (with slight variations) constant in all groups (Table I).

Testis (T) shows shrinkage of the semeniferous tubules (ST) in Pb group with a simultaneous damage to the interstitial tissues leaving wider gaps among the tubules as compared to the Cont group while rehabilitation signs of the interstitial tissue are recognizable in Pb-J group. Overall shrinkage of Corpus EP and lesser number of folding tubing are visible in Pb than Cont and Pb-J groups. Vas Deference (VD) in Cont group shows thick adventitia and muscularis layers with the mucosa containing longitudinal folds (cristae) and the lumen harbor scanty sperms. The cristae are not recognizable while the lumen of the VD appears fully gorged with sperm debris in Pb and Pb-J groups.

**Figure 3:**
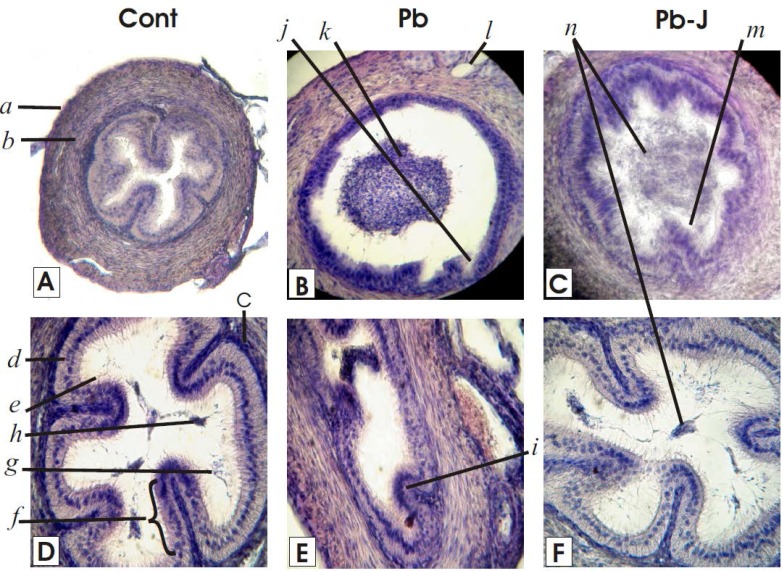
Histological sections of vas deference in control, Pb and Pb-J group mice (A, B and C: 400×; D, E and F 1000×)

Control (Cont) group sections show well developed adventitia (a) and muscularis (b). The lamina propria appeared as a thick basement membrane (c) while the endothelium contained columnar principal cells (d) that bore stereocillia towards the lumem (e). The endothelium was frequently thrown into stereovilli (f). The lumen contained only a few mature spermatozoa (g) and a little debris (h).Lead ( Pb) group slides show flatting of the endothelial lining consisting of pseudo-stratification of the somewhat cuboidal principal cells with only a few stereocillia and slight remnants of stereovilli (i) where as some endothelial lesions are also visible (j). The rounded luminal space contains central clumps of spermatic mass (k). The muscularis show signs of fibrosis (l).

The Pb-J group slides indicate partial rehabilitation of the stereocillia and stereovilli (m) of the endothelium. Diffused clumps of spermatic cell mass (n) are visible in the luminal space. Partial regeneration of the muscularis with no prominent signs of fibrosis as seen in the Pb group along with the rehabilitation of lamina propria and adventitia are clearly recognizable.

**Table I:** Micrometric results for cross sectional area and the thickness of the epithelial cell lining of the epididymis

**Organs**	**Parameters**	**Groups**		
		**Cont**	**Pb**	**Pb-J**
Caput epididymis folding tubing	CSA µ^2^	‡ 8236.71± 464.5^a^	7835.84±428.7^b^	8031.05±482.7 a
	EH µ	23.3962±1.2^a^	19.4203±0.8^b^	24.3595±0.7^a^
Corpus epididymis folding tubing	CSA µ^2^	12642.75±1423.3^a^	16448.35±762.7^b^	11739.21±1590.3^c^
	EH µ	22.68±0.7^a^	19.57±0.6^b^	20.99±0.6^c^
Cauda epididymis folding tubing	CSA µ^2^	13717.6±1174.6^ab^	12448.5±762.7^a^	14739.2±1590.3^b^
	EH µ	19.0027±0.7^a^	20.1998±0.6^a^	18.5435±0.5^a^

Cont: Control, Pb: 50ppm Pb, Pb-J: jambul pulp extract post treatment of Pb exposure, CSA; Cross sectional area, EH; Epithelial height/thickness, µ; micrometer, ±; standard deviation,

‡ **; **average of 10 folding tubing,

abc ; indicate variation among groups, ANOVA; Analysis Of Variance, Duncan multiple range test. Groups share a common lowercase alphabet, do not vary significantly, as indicated by Duncan multiple range test (p≤0.05).

**Vas deferens**

Control Group: The histological study of the vas deferens in the control group at 400 and 1000× indicated outer denser covering of adventitia. The muscular layer was well developed and consisted of smooth thick longitudinal and thin inner layers which made vas deferens to show peristaltic contractions for the movement of sperms. The mucosa of the vas deferens in control group slides consisted on lamina propria containing rich elastic fibers and the columnar ciliated endothelium that formed longitudinal folds (like cristae of mitochondria) in the lumen called microvilli (stereovilli). The lumen contained only a few spermatozoa (Figure 3.A and 3.D). *Lead (Pb) Group:* In Pb group the vas deferens had shown various histo-pathologies that include disappearance of cristae like folding of the mucosa and widened rounded to ellipsoidal luminal space (depending on the shape of the tubing) the endothelial lining of the mucosa showed pseudo-stratification of cuboidal to squamous endothelium while the lamina propria was inconspicuous. The stereocilia in most of the histological slides in Pb exposed group were not clearly visible. The microvilli (stereovilli) were apparently smashed. The endothelium lining has shown various lesions (cracks) at various places. The lumen contained clumps of spermatids amd short-tailed sperms in the center. The musculature and adventitia were less developed and often showed fibrosis of the musculature (Figure 3.B and 3.E).

Lead- Jambul (Pb-J) Group: In Pb-J sections the mucosa of the vas deferens consisted on pseudo-stratification of the slightly elongated cuboidal endothelial principal cells with scanty stereocillia. The lamina propria also showed slight elevations indicating the beginning of reappearance of the stereovilli. The lamina propria also seemed to the rehabilitated. The muscularis was thicker than Pb group additionally it also showed far lesser fibrosis that that of the Pb group slides. Lumen contained less denser spermatic mass than Pb group (Figure 3.C and 3.F).

## Discussion

Histopatholgical ameliorations of heavy metals are not limited to liver, kidney, endocrine gland and reproductive organs, but also left long lasting sweeping effects on every exposed living part (22). Besides testis, the associated reproductive organs such as epididymis, vas deferens, seminal vesicle and prostate are equally important for the male reproductive health. Whereas the primary job of testis is to produce sperm cells and male sex hormones (principally testosterone). The associated organs are required to nurture and help to bring about maturational changes; storage, maintenance of health and sperm transport along with the production of their particular secretions into the ejaculated semen. These activities are rather important for sexual vigor and potency in the males. The stereocilia in the epididymis and vas deferens are more like the long, absorptive microvilli, which increase the surface area of the cell and aid in absorption and secretion. Because sperm cells are initially non-motile when they leave the seminiferous tubules, enter the caput epididymis, progress to the corpus, and finally reach the cauda region, where they are stored temporarily. 

During transit in the epididymis the spermatozoa undergo maturational changes. The stereocilia of the principal cells create a fluid current that moves the immobile sperm from seminiferous tubules to the epididymis. This transport maybe interrupted by metal induced apoptosis of the principal cells (23-24). 

The results in the present study specified various histologic and micrometric changes characteristic to the three distinct regions (caput, corpus, and cauda) of epididymis and vas deferens (Figure 2, 3; Table I). 

Hypertrophy of the principal cells and their associated stereocilia and signs of phagocytosis, particularly in caput and corpus region clearly indicate their active role in the removal of dead and abnormal sperms released from testes on Pb exposure (25). In present study the disruption of endothelium with pycnotic cell nuclei, clumping of stereocilia, reduction in sperm density and cellular debris in the lumen were the major histopathological alterations of the epidiydymis. On the other hand Pb deposition in the walls of the seminiferous tubules and suppression of the hypothalamic-pituitary-testicular axis are believed to cause testicular histological alterations and lowering of circulating testosterone (9). Pb exposure had also showed degenerated interstitial layer, empty tubing, and thickening of a muscular layer similar to cadmium exposure (26). 

The reduction of protein level by metal exposure in the caput and cauda epididymis intimated inhibition of enzymes and secretions to interrupt the mitochondrial physiology and oxidative metabolism (27). The energy deficient cell indicated lipogenesis and ultimately suffered from vacuolation and apoptosis. The bulging of tubing evident by elevation of cross-sectional area directed intra-tubing material pressure potential, which dilated the epithelial thickness and damage stereocilia (Figure 2 and 3; Table I). Thick epithelium with lager nuclei indicated the metallic effect on the mitochondrial functions and inhibition of ATPase enzymes activity, which may also cause cellular shrinkage secondarily (28). The bulk of spermatozoa in the central area of vas deferens can be co-related to the destruction of stereovilli (Figure 3.B). Polyphenols are potent antioxidants because of their ability to donate hydrogen and thus help to quench singlet oxygen (^1/2^O_2_) and chelate metal ions (10-12). Jambul fruit pulp has excellent antioxidant properties and has free radical scavenging potential (16). Treatment with jambul pulp extract after Pb exposure in the present study, helped tissue recovery by virtue of its anti-oxidant properties and additionally due to its probable role in increasing cyclic- Adenosine monophosphate levels- restoring enzyme activities, metabolism and growth (27). 

**Figure 4:**
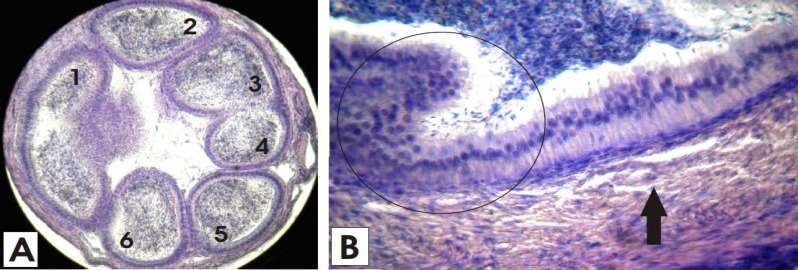
Epididymis and vas deferens regeneration in mice

Reversal of these signs of apoptosis in pseudostratified endothelium with a simultaneous increased distribution of sperm density specified the rehabilitation of the epididymis tubing (Figure 4.A and 4.B).

Syzigium cumini and Vitamin C attenuate the similar effects in a study about induced nephrotoxicity and alterations in renal brush border membranes (29). The large size tubing in epididymis when damaged may constrict like binary fission furrow and change into smaller tubing during regeneration after proliferation. The larger tubing are more vulnerable and thus adversely affected while smaller tubings are apparently more rounded and stable (Figure 4.A). 

Based these results, Pb exposure has been found to cause various types of structural alterations in the epididymis and vas deferens while Jambul has been found to invalidate such compensatory changes occurring in seminiferous tubules in testis (20). Jambul has the ability to remove noxious materials through its scavenging ability and inhibition of oxidation of molecules to prevent the body from oxidative stress that leads to various tissue pathologies. It combats with the free radicals and prevent body from lipid peroxidation by up-regulating enzymes during ameliorative activities. The other possible mechanism of Jambul to mitigate the Pb toxicity may be through up-regulating the peroxisome proliferator-activated receptors and membrane phospholipid. These findings are in accordance with the previous results and data that support the validity of our model and confirm the recommendation about Jambul as anti-oxidative, anti-apoptotic and regenerative properties in epididymis and vas deferens. 

## Conclusion

Conclusively, it can be stated that Pb exposure causes various histopatholgical changes in epididymis while Jambul extract has been found to normalize all such histological and micrometric signs indicating its vital role in the health of reproductive organs.

**A:** Epididymis (Folding Tubing 1-6), **B:** Vas Deferens, 1; Folding Tubing regeneration and proliferation, 3-4; represent regenerated Folding Tubing, circle indicates the proliferation and regeneration, black arrow indicate lamina propria rearrangement and regeneration of the muscularis.

## Note

***This article extracted from M.Sc. thesis. (Samreen Iqbal)***

## References

[1] Freer-Smith P, Carnus JM (2008). The sustainable management and protection of forests analysis of the current position globally. Ambio.

[2] Yang YQ, Chen FR, Zhang L, Liu J, Wu S, Kang M (2012). Comprehensive assessment of heavy metal contamination in sediment of the Pearl River Estuary and adjacent shelf. Mar Pollut Bull.

[3] Florea AM, Busselberg D (2006). Occurrence use and potential toxic effects of metals and metal compounds. Biometals.

[4] Mahdy TM, Giorgi T, Adewole F, Ernest I, Idoko M, Matey N (2012). Effect of Moringa oleifera, activated carbon and wood charcoal on biochemical and hematological parameters of Wistar rats exposed to lead acetate. Med Weter.

[5] De Marco M, Halpern R, Barros HM (2005). Early behavioral effects of lead perinatal exposure in rat pups. J Toxicol.

[6] Pizzol M, Thomsen M, Anderson MS (2010). Long-term Human Exposure to Lead from Different Media and intake Pathways. Sci Total Environ.

[7] Kojima M, Sekikawa K, Nemoto K, Degawa M (2005). Tumor Necrosis Factor-α-Independent down regulation of hepatic cholesterol 7α-Hydroxylase gene in mice treated with Lead Nitrate. Toxicol Sci.

[8] Bomfim KMA, Garcia CAB, Reis FP, Palmeira RJAV, Scher R, Aragao JA (2012). Absorption levels and morphological features of fetal organs in wistar rats treated with lead acetate. Int J Morphol.

[9] Hamadouche NA, Sadi N, Kharoubi O, Slimani M, Aoues A (2013). The protective effect of Vitamin E against genotoxicity of Lead Acetate intraperitoneal administration in male rat. Arch Biol Sci Belgrade.

[10] Lie C, Xie B (2000). Evaluation of the antioxidant pro-oxidanteffects of tea catechin oxypolymers. J Agric Food Chem.

[11] Gulcin I (2005). The antioxidant and radical scavenging activities of black pepper seeds. Int J Food Sci Nutr.

[12] Gebicka L, Banasiak E (2009). Flavonoids as reductants of ferryl hemo- globin. Acta Biochim Pol.

[13] Yadav RNS, Agarwala M (2011). Phytochemical analysis of some medicinal plants. J Phytol.

[14] Moshi MJ, Otieno DF, Weisheit A (2012). Ethnomedicine of the Kagera Region, North Western Tanzania. Part 3: plants used in traditional medicine in Kikuku village, Muleba District. J Ethnobiol Ethnome.

[15] Abdel-Hameed ES, Bazaid SA, Shohayeb MM, El-Sayed MM, El-Wakil EA (2012). Phytochemical studies and evaluation of antioxidant, anticancer and antimicrobial properties of Conocarpus erectus L. growing in Taif, Saudi Arabia. Europ J Med Plants.

[16] Gordon A, Jungfer E, Da-Silva BA, Maia JG, Marx F (2011). Phenolic constituents and antioxidant capacity of four underutilized fruits from the amazonregion. J Agric Food Chem.

[17] Venkateswarlu G (1952). On the nature of the colouring matter of the jambul fruit (Eugenia jambolana). J Indian Chem Soc.

[18] Sengupta P, Das PB (1965). Terpenoids and related compunds part IV triterpenoids the stem-bark of Eugenia jambolana Lam. Indian Chem Soc.

[19] Soncharan P, Shanmugarajan TS, Somasundaram, Niladri M (2010). Protective effect of Syzygium cumini seeds against doxorubicin induced cardiotoxicity in rats. Int J Phar Life Sci.

[20] Ahmad KR, Nauroze T, Raees K, Abbas T, Kanwal MA, Noor S (2012). Protective role of jambul (Syzygium cumini) fruit-pulp extract against fluoride-induced toxicity in mice testis: a histopathologiocal study. Flouride.

[21] Bancroft JD, Gamble M (2003). Theory and Practice of histological techniques.

[22] Bharali MR (2013). Effect of acute lead acetate exposure on liver of mice. Journal of Global Biosciences.

[23] Xin-hong L, Zhi-ying W, Li Qin Zhen-liang C, Xu Y (2010). Effects of cadmium on testis spermatocyte apoptosis and epididymis mature spermatozoa quality of mice. Acad J.

[24] Shum WW, Smith TB, Cortez-Retamozo V, Grigoryeva LS, Roy JW, Hill E (2014). Epithelial basal cells are distinct from dendritic cells and macrophages in the mouse epididymis. Biolo Reprod.

[25] Sharma R, Garu U (2011). Effects of lead toxicity on developing testes in Swiss mice. Univ J Environ Res Technol.

[26] Singh P, Deora K, Bano H, Mogra P, Javeria S, Barolia S (2012). Protective effect of curcumin on cadmium chloride induced epididymal toxicity in Swiss mice (Mus musculus). J Chem Bio Phy Sci Sec.

[27] Chinoy NJ, Momin R, Jhala DD (2005). Fluoride and Aluminium induced toxicity in Mice Epididymis and its mitigation by Vitamin C. Fluoride.

[28] Young-Ok SJ, Hitron A, Wang X, Chang Q, Pan J (2010). Cr (VI) induces mitochondrial-mediated and caspase-dependent apoptosis through reactive oxygen species-mediated p53 activation in JB6 Cl41 cells. Toxicol App Pharmacol.

[29] Fatima S, Mahmood R (2007). Vitamin C attenuates potassium dichromate-induced nephrotoxicity and alterations in renal brush border membrane enzymes and phosphate transport in rats. Clin Chim Acta.

